# Investigation of the Mechanisms of Transition of Gram-Negative Bacterial Cells into Induced Anabiosis Using Computational Methods of Classical Molecular Dynamics

**DOI:** 10.3390/microorganisms14020472

**Published:** 2026-02-14

**Authors:** Ksenia Tereshkina, Eduard Tereshkin, Licheng Zhang, Petr Zaytsev, Vladislav Kovalenko, Yuriy Litti, Olga S. Sokolova, Yurii Krupyanskii, Nataliya Loiko

**Affiliations:** 1 Semenov Federal Research Center for Chemical Physics, Russian Academy of Sciences, 119991 Moscow, Russia; ramm@mail.ru (E.T.); yuriifkru@gmail.com (Y.K.); 2 Faculty of Biology, Lomonosov Moscow State University, 119234 Moscow, Russia; 3 Winogradsky Institute of Microbiology, Fundamentals of Biotechnology Federal Research Center, Russian Academy of Sciences, 117312 Moscow, Russia; litty-yuriy@mail.ru

**Keywords:** *Escherichia coli*, dormant cells, bacterial cell survival, classical molecular dynamics, membrane of Gram-negative cells, 4-hexylresorcinol, bacterial antibiotic resistance

## Abstract

Studying the mechanisms by which Gram-negative heterotrophic bacteria transition from active metabolism to dormancy is an important task, as it is directly related to the problem of bacterial antibiotic resistance and the spread of nosocomial infections. Using electron microscopy, microbiology, and molecular modeling, we investigated the dose-dependent mechanisms of action of 4-hexylresorcinol (4HR), a chemical analog of the anabiosis autoinducer, on the cell membranes of Gram-negative bacteria (using *Escherichia coli* as an example), leading to the formation of stressed, dormant, and mummified cells. It was shown that 4HR penetrates membranes equally easily both as single molecules and as micelles, distributing itself across the membrane so that the hydrocarbon radicals are aligned parallel to the lipid tails. When micelles penetrate the membrane, uneven distribution of 4HR within and between leaflets occurs, as well as lipid redistribution within the membrane, leading to the appearance of a third peak on the phospholipid electron density profile and a third black band in the membrane region in TEM images of such cells. At 4HR concentrations in solution of 200 µM, its micelles cover the cell membranes in a thick layer, penetrate into the membrane, and completely saturate it. Even higher concentrations create agglomerates or actually micellar arrays within the cell membranes, leading to cell death through mummification.

## 1. Introduction

In nature, microorganisms inhabit small ecological niches where periods of active growth alternate with periods of dormancy characterized by the absence of cellular metabolism [1]. These constant transitions are caused by changing environmental conditions, a lack of nutrient substrates, fluctuations in abiotic factors during seasonal changes. Typically, dormant periods of microorganisms are longer than periods of active life and can last for years [2]. To cope with unfavorable conditions and survive, microorganisms have developed adaptive strategies that allow them to quickly enter a dormant state [3].

In Gram-negative bacteria, including *Escherichia coli*, starvation stress during the stationary phase triggers the alternative sigma factor RpoS, which controls up to 10% of all cellular genes [4]. And gradually part of the bacterial population passes into another functional state, forming the so-called anabiotic or dormant cells (DCs) [5]. Such DCs do not exhibit metabolic activity and have increased resistance to prolonged incubation, as well as to many stress factors, for example, changes in temperature, pH, salinity, the effects of antibiotics or other biocides [6]. Moreover, DCs are heterogeneous and differ in the degree of stability and the depth of dormancy, which provides the population with the ability to quickly switch to growth when favorable conditions arise (due to less “dormant” DC) or to survive particularly severe stress (due to strongly restructured DCs) [6,7]. The transition to a dormant state is accompanied by significant changes in cell morphology, therefore the structure of DCs differs greatly from the structure of actively dividing cells [8]. In DCs, the protoplast is transformed, the cell wall changes, DNA is compacted with the help of nucleoid proteins, primarily Dps [7,9]. The changes also affect cell membranes, which become denser [10].

The transition to a dormant state of Gram-negative heterotrophic bacteria, which include pathogens such as *E. coli* K1 (causes meningitis and sepsis), *Pseudomonas aeruginosa* (causative agent of nosocomial infections), *Acinetobacter baumannii* (causative agent of nosocomial infections), *Klebsiella pneumoniae* (causative agent of pneumonia, sepsis, uri-nary tract infection), *Proteus vulgaris* (causes wound infections), *Treponema pallidum* (causative agent of syphilis), *Borrelia burgdorferi* (causative agent of Lyme disease (borreliosis)) is directly related to the problem of bacterial antibiotic resistance and the spread of nosocomial infections [11]. In a dormant state, pathogenic bacteria stop the synthesis of proteins and peptide glycans, as well as other metabolic processes that are targets of various antibiotics [12]. Therefore, dormant pathogenic cells become resistant to the action of anti-biotics. In this state, they can persist in the human body for a long time or be preserved on hospital objects [13]. The ability to influence the functional state of bacteria and control their transitions to a dormant state and back will allow us to find new tactics in the treatment of infectious diseases, especially chronic infections [12].

Effective management of bacterial dormancy will enable microbial collection specialists to preserve sensitive bacteria for which standard cryopreservation and lyophilization methods result in fatal cell damage and death [14]. This is also important for maintaining collections of biotechnologically important bacterial strains in industrial facilities and agriculture [15]. However, despite the importance of this issue for both medicine and biotechnology, it remains extremely poorly understood.

The transition of bacteria to a dormant state is carried out by molecular mechanisms including translational and transcriptional regulation triggered by signaling molecules [16]. In the works of El-Registan et al., it was shown that the processes of DC formation in bacterial populations are regulated, among other things, by special signaling molecules—autoinducers of anabiosis, secreted by all cells of the population at late stages of growth [17,18]. These signaling molecules were found in Gram-positive and Gram-negative bacteria of various species [17,18,19,20]. It turned out that they belong to the class of alkylresorcinols [17,21]. An increase in the level of these autoinducers in an econiche or a laboratory flask led to an increase in the number of DC formed in the population [22]. To study the mechanisms of this phenomenon, a chemical substance—4-hexylresorcinol (4HR)—was selected, which acted on bacterial cells of different species similarly to native autoinducers of anabiosis isolated directly from cell populations [17,22,23,24]. The addition of a chemical analog of the anabiotic autoinducer 4HR to a population of different bacterial species in the pre-stationary or stationary phase at a certain concentration caused an almost instantaneous change in the functional state of the cells and their transition to a dormant state [25]. The effect of 4HR was dose-dependent. Low concentrations (1–100 μM) increased the stress resistance of the cells. Higher concentrations (approximately 200 μM) caused rapid transition of cells into an anabiotic state, and increasing the exposure time or concentration of 4HR led to cell death by mummification [26,27]. Although cells in an anabiotic state do not exhibit metabolic signs of life, they remain viable for a long time. After certain reactivation procedures, such cells return to active life, allowing the population to survive under the most unfavorable conditions. In contrast, mummified cells exposed to a supercritical dose of 4HR die, although they retain their morphological structure, being saturated with 4HR. This effect is caused by the fact that 4HR can interact not only with membrane lipids but also penetrate into the cell and interact with all cellular biopolymers [28,29]. No reactivation procedures can revive mummified cells.

It turned out that the mechanisms of DC formation, with the help of 4HR, are very similar to the mechanisms of DC formation under native conditions during starvation, therefore their study can shed light on general issues of cell dormancy [30]. However, studying them using experimental biological methods is a difficult and sometimes impossible task. In such cases, computational biology methods come to the rescue. In previous studies, we demonstrated the effectiveness of classical molecular dynamics (MD) methods in studying various biological processes and mechanisms occurring in vivo in living cells or in vitro with cellular biopolymers [31,32]. Simulations performed using classical and steered molecular dynamics methods showed that 4HR is capable of penetrating Gram-negative bacterial cells through lipid bilayers of membranes and directly affecting lipopolysaccharides, proteins, peptidoglycan, and DNA [32,33]. However, the problem of 4HR interactions with Gram-negative bacteria, leading to the formation of stress-resistant, dormant, or mummified forms, still leaves more questions than answers.

The aim of this study was to conduct a detailed investigation of the effect of 4HR on the membranes of Gram-negative bacterial cells using microbiological, microscopic, and classical MD methods. The objectives of the study were to answer the following questions:

1. How can 4HR, a poorly water-soluble substance, penetrate bacterial cells when its alcohol solutions are added to cell suspensions?
2. How does 4HR affect the cell wall of Gram-negative bacteria? Specifically: (1) How is 4HR integrated into the lipid bilayer of bacterial membranes, distributed there, and what happens to the lipid bilayer itself? (2) How does this process depend on the concentration of the added 4HR? (3) How does 4HR affect the peptide-glycan layer?
3. Why is the concentration range (150–250) µM 4HR a threshold for the transition of cells from a stress state through a dormant state to a mummified state?

## 2. Materials and Methods

***Microbiological methods***

### 2.1. Bacterial Strain and 4HR

The objects of the study were the Gram-negative bacteria *Escherichia coli* Top10 from the Biotechnology Research Center collection [6,9].

4HR was purchased from Sigma-Aldrich (St. Louis, MO, USA). Immediately before the experiment, a 0.1 M ethanol solution of 4HR was prepared by dissolving 20 mg of 4HR in 1 mL of 96% ethanol. The remaining ethanol solutions of 4HR were prepared from this solution by dilution. Ethanol solutions of 4HR were added to the cell suspensions to the desired concentration so that the final ethanol concentration was 1%.

### 2.2. Cultivation

Bacteria were grown in 250 mL cotton-stoppered flasks containing 50 mL of Luria–Bertani (LB) medium (Miller’s LB Broth, VWR, Radnor, PA, USA) with shaking (140 rpm) and a temperature of 28 °C. Bacterial populations were stored in plastic falcons at 23 °C for 16 months with periodic determination of viable cell counts [6,9].

The growth of bacterial populations was controlled by the change in the optical density of the suspension, which was measured at 595 nm on a Spectrophotometer 7315 (Jenway, Stone, Staffordshire, UK).

Cell viability was determined by counting colony-forming units (CFU) after plating diluted cell suspensions on LB agar medium (with the addition of 3% Bacteriological Agar, Helicon, Moscow, Russia).

### 2.3. Experiments on the Effect of 4HR on Bacterial Cells

To study the effect of 4HR on bacterial cells, ethanolic solutions of the preparation were added to *E. coli* bacterial populations in the pre-stationary growth phase to final concentrations of 10, 100, 200, and 1000 µM and incubated without stirring at 23 °C. Equivalent amounts of ethanol were added to the control populations. The resulting experimental populations were used in microbiological and microscopic experiments.

The number of viable cells in the experimental populations was assessed periodically after 40 min, 1 day, 7 days, 1 month, and 16 months of incubation by plating on solid media, as described previously. In some cases, a reactivation procedure was performed before determining the number of viable cells. To do this, an aliquot of the bacterial population was placed in a flask with sterile water at a ratio of 1:100 and incubated for 24 h with constant stirring at a temperature of 23 °C. The cell suspension was then concentrated, and the number of viable cells was determined as described previously.

### 2.4. Study of the Thermal Stability of Bacterial Cells

The thermostability of bacterial cells was determined by their survival after a warm-up of cell suspension (450 µL) for 20 min in a thermoshaker (BIOSAN TS_100, 450 rpm, Riga, Latvia) at a temperature of 50 and 60 °C, followed by determining the number of viable cells by counting CFU on an LB agar plate.

The titer of surviving cells was calculated by finding the ratio of the number of surviving cells to their initial number before thermal exposure and expressing this ratio as a percentage.

***Microscopic methods***

### 2.5. Obtaining Images Using a Light Microscope

Microscopic observations were carried out using a ZEISS Axio Imager M2 microscope (Göttingen, Germany) with a phase-contrast device.

### 2.6. Transmission Electron Microscopy (TEM)

Cells were fixed with 2% glutaraldehyde for 5 h and, after re-fixation with 0.5% paraformaldehyde, washed with 0.1 M cacodylate buffer (pH 7.4), counterstained with 1% OsO4 in cacodylate buffer (pH = 7.4), dehydrated in an increasing series of ethanol solutions followed by desiccation with acetone, impregnated and embedded in Epon-812 (according to the manufacturer’s instructions). Ultrathin sections (70–200 nm thick) were cut with a diamond knife (diatom) on an Ultracut-UCT ultramicrotome (Leica Microsystems), transferred to 200 mesh copper grids coated with Formvar (SPI, USA) and counterstained with lead citrate, according to the established Reynolds procedure [34].

Ultrathin cell sections were examined in a JEM-2100 transmission electron microscope (Jeol, Tokyo, Japan) with an accelerating voltage of 200 kV and a magnification of ×13,000–21,000. Images were recorded using Ultrascan 1000XP and ES500W CCD cameras (Gatan, Pleasanton, CA, USA).

***Classical MD methods***

### 2.7. Bilayer and Peptidoglycan Models

Stable in silico models of the bacterial membranes consisting of 1-palmitoyl-2-oleoyl-sn-glycero-3-phosphoethanolamine (POPE) and 1-palmitoyl-2-oleoyl-sn-glycerol-3-phosphoglycerol (POPG) molecules in a 3:1 ratio were generated using CHARMM-GUI Membrane Builder [35]. Simulation boxes contained the bilayers, Extended Simple Point Charge water molecules (SPC/E water), ions, and 4-hexylresorcinol (4HR) molecules. All studied membranes had the same initial volume ~450 nm^3^ of periodic boxes, 62 Na^+^ and 20 Cl^−^ ions, about 40 thousand atoms with different ratios of 4HR to lipids molecules (Table 1). The systems were subjected to energy minimization followed by constant volume relaxation and a series of constant pressure relaxations according to a standard CHARMM-GUI protocol. Force field parameters for 4HR were found using ab initio calculations with FIREFLY (PC GAMESS) 8.2.0 quantum-chemical software package [36]. *Insert-molecules* tool of GROMACS [37] was used to insert 4HR molecules into a solvated configuration of membrane systems, where solvent atoms were replaced with the 4HR atoms. We simulated three trajectories from different starting configurations for each 4HR concentration to reduce potential bias from the initial configuration.

The peptidoglycan model consisted of two strands of alternating sugar residues β-1,4-linked N-acetylglucosamine and N-acetylmuramic acid. Two cross-peptide links and 6 pentapeptides (L-Ala, γ-D-Glu, meso-A_2_pm, D-Ala, D-Ala) attached to N-acetylmuramic acid were modeled per 20 sugar residues. In a periodic box of ~1180 nm^3^ 100 molecules of 4HR, 151 Na^+^, 29 K^+^, 162 Cl^−^, 1 Ca^2+^ and 37,220 SPC/E water molecules were added to the system.

To comprehensively study the effects of 4HR on membranes, several types of modeling were conducted. The adsorption of 4HR molecules on membranes, the incorporation of individual molecules, and the dissolution of 4HR vesicles in membranes at zero surface tension were investigated.

### 2.8. MD Simulations

Molecular dynamics simulations were performed with Gromacs 2024.4 using the AMBER99-PARMBSC1 all-atom force field [38]. A Langevin thermostat [39] set to 310 K = 37 °C with an inverse friction constant of 2 ps was used to maintain constant temperature. The isothermal compressibility of water was set to 4.5·10^−5^ bar^−1^. A three-dimensional periodic simulation box was used. Bonds to hydrogen atoms were constrained using the parallel version of LINEar Constraint Solver (P-LINCS) [40]. Particle mesh Ewald (PME) [41] long-range electrostatic interactions and a buffered neighbor list according to the Verlet scheme were implemented. Cutoff distances for electrostatic, van der Waals, and neighbor interactions were set to 1.5 nm. The membranes were simulated with a time step of 2 femtoseconds for 50–1000 nanoseconds (ns) depending on simulation.

The surface tension coupling in the x/y directions (from 0 to 40 mN/m) and the pressure coupling in the z direction (at 1 atm and a coupling constant of 0.5 ps^−1^) were simulated using the C-rescale barostat. In this paper, unless otherwise noted, conventional simulations are performed at zero surface tension. For the peptidoglycan simulations, the pressure was maintained semi-isotropically at 1 atm and a coupling constant of 0.5 ps^−1^ independently in the x/y and z directions.

The simulation of surface tension increase started with membranes pre-equilibrated for 100 ns and was increased by 5–10 mN/m every 50 ns. A similar membrane stretching protocol was developed previously [42] and showed stable results for bilayers at the surface tension values used here.

## 3. Results

### 3.1. Effect of 4HR on the Survival and Stress Resistance of Bacterial Cells: Formation of Stressed, Dormant, and Mummified Cells Under the Influence of 4HR

The study focused on the Gram-negative bacteria *E. coli* Top10 [6,9]. To study the effect of 4HR on bacterial cells, solutions of various concentrations were prepared in 96% ethyl alcohol. The solutions were added to cell suspensions to achieve the desired 4HR concentration, resulting in a final alcohol concentration of 1%. 4HR, which is poorly soluble in water (<0.1 g/100 mL), formed micelles, which manifested as turbidity in the solutions.

**Figure 1:**
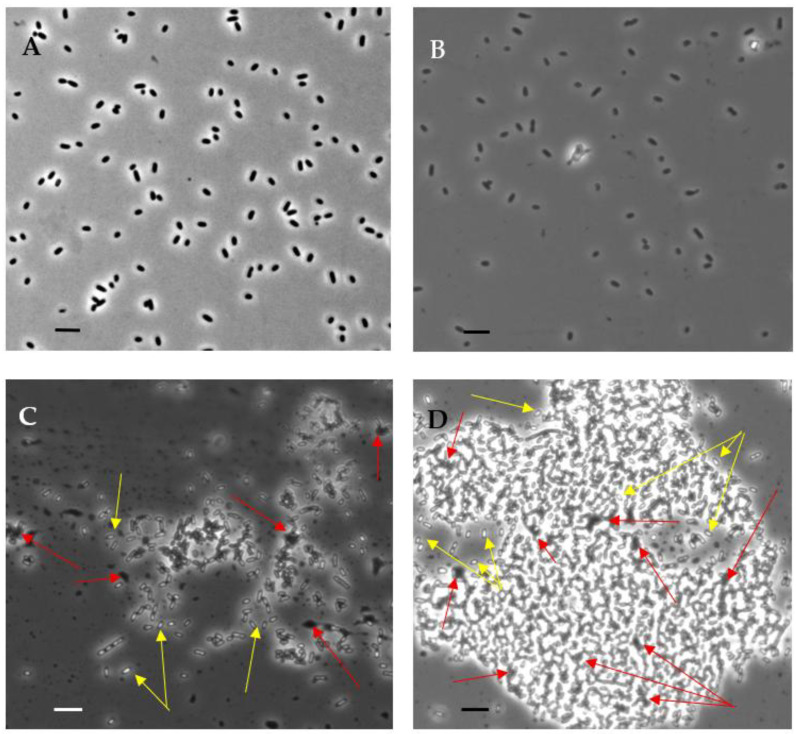
*E. coli* Top cells observed by phase-contrast microscopy: (**A**)—control stationary cells; (**B**–**D**)—stationary cells that were exposed to 4HR for 40 min at concentrations of 100, 200 and 1000 µM, respectively. Red arrows indicate areas of cell lysis; yellow arrows indicate cells that refract light most strongly. Scale bar—5 µm.

The introduction of small doses of 4HR up to concentrations of 100 µM inclusive into bacterial populations at the beginning of the stationary growth phase did not cause noticeable changes in the cell morphology (shape and size) compared to the control bacteria when examined under a light microscope with phase contrast at a magnification of 1100× (Figure 1A,B). The number of viable cells in the population also remained virtually unchanged (Table 2). However, doubling the concentration of introduced 4HR to 200 µM led to noticeable changes after just 40–60 min. Some of the cells in the population died (zones of cell lysis appeared), while the cytoplasmic structure of other cells changed. These cells began to refract light more strongly, acquiring an appearance similar to spores (when examined in phase contrast) (Figure 1B). The number of viable cells decreased by three times after 40 min of exposure to 4HR. Further increasing the 4HR concentration by two or more times led to a more pronounced effect. The number of dead cells increased, and any remaining cells shrank in size and strongly refracted light (Figure 1D). The viability of such cells was no longer detectable by the methods used, even after reactivation procedures (i.e., washing off 4HR) (Table 2).

During further long-term (16 months) incubation of the experimental populations, cell survival trends diverged. In the experimental variants with concentrations of 10 and 100 µM, the presence of 4HR promoted the survival of a greater number of bacteria than in the control. In the variant with the addition of 200 µM 4HR, no viable cells were detected after just one month of incubation without special reactivation procedures. After 16 months, even after these procedures, no viable cells were detected (Table 2). It is likely that the surviving DCs in these populations entered a very deep dormant state.

It should be noted that similar patterns were also observed when working with other *E. coli* strains and in experiments with other Gram-negative heterotrophic bacteria, for example, *Pseudomonas aeruginosa* 209P (these data are not provided in this study) [30,32]. In each case, it turned out that it was the concentration range (150–250) µM that caused a rapid transition of bacterial Gram-negative cells of different species to a dormant state with a change in their morphology. However, long-term storage of such DC led to the immersion of most cells into a very deep dormancy, from which it was difficult for them to return to an active state. Doses of 4HR that were two times (or more) smaller caused the formation of a population of stressed cells, which became resistant both to long-term storage without loss of viability and to the effects of stress factors, such as thermal shock: exposure of cells to temperatures of 50 and 60 °C for 20 min. The results of these experiments are presented in Table 3. Doses of 4HR that were twice as high (or more) resulted in extreme changes in cells, incompatible with their viability. In such populations, cells that did not immediately die or lyse transitioned, in less than 40 min, to a so-called mummified state, characterized by complete preservation of cellular integrity but complete loss of viability.

### 3.2. Changes in the Ultrastructure of Bacterial Cells Exposed to 4HR

**Figure 2:**
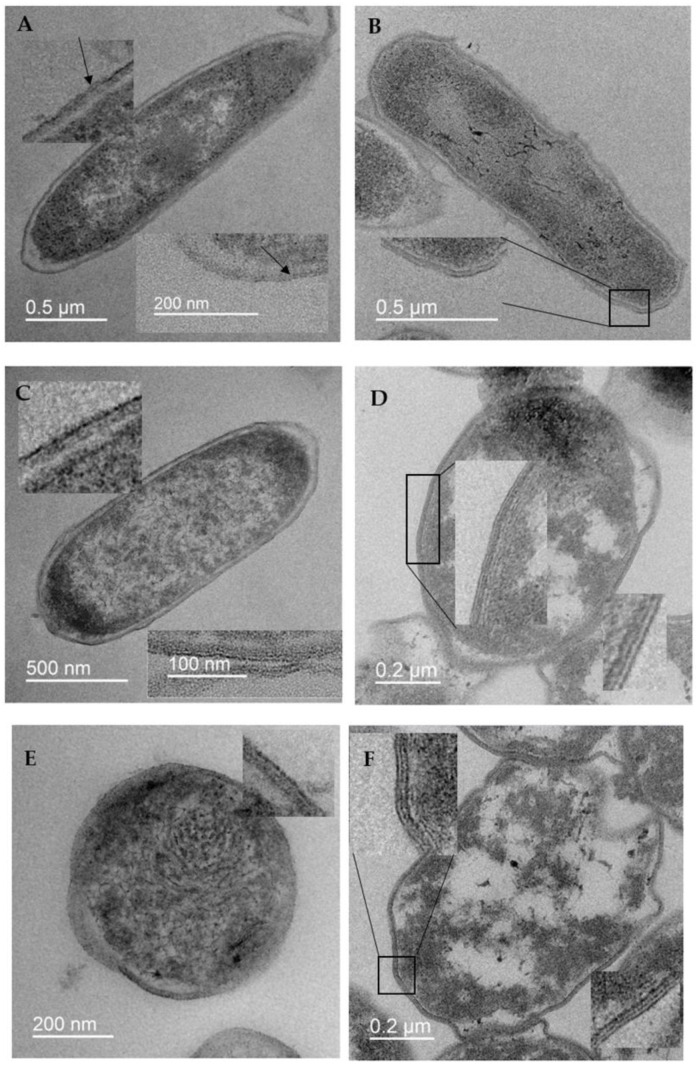

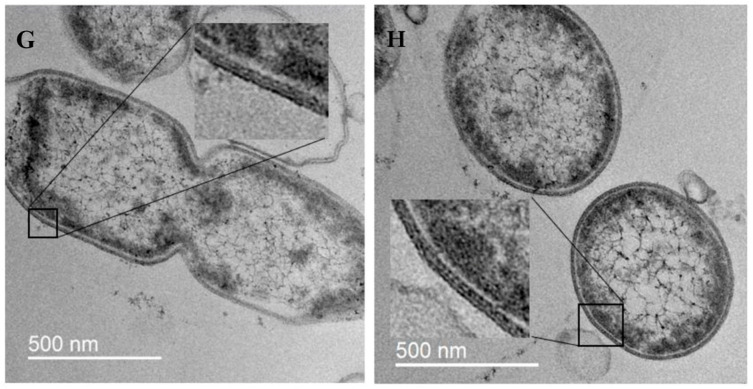
Electron micrographs of *E. coli* cells incubated for 1 day (**A**,**C**,**E**,**G**) and 16 months (**B**,**D**,**F**,**H**): (**A**,**B**)—stationary cells; (**C**,**D**)—cell obtained after exposure to 4HR of 100 µM; (**E**,**F**)—200 µM; (**G**,**H**)—1000 µM. The figures have been inset with high magnification.

The ultrastructure of stressed, resting, and mummified cells incubated for 24 h and 16 months, obtained after exposure to different concentrations of 4HR, was studied using transmission electron microscopy (TEM). The focus was on comparing changes in the cell membranes (Figure 2).

In TEM images of stationary cells, both those incubated for 24 h and those incubated for 16 months, the outer and inner (barely visible) membranes appeared as two dark lines with a lighter peptide glycan band in the middle (Figure 2A,B). The thickness of the outer membrane of a daily stationary cell was approximately 4.5 nm, while that of a cell stored for 16 months was approximately 8 nm. The membranes of cells exposed to 4HR looked different; a third line appeared next to the two dark lines (Figure 2C–H). Moreover, the higher the 4HR concentration, the more noticeable the third dark line. In images of cells exposed to a low 4HR concentration of 100 µM for 24 h, the membrane had three dark lines only in places (Figure 2C). Meanwhile, in cells incubated for 16 months, the membrane had three dark lines almost everywhere (Figure 2D). This membrane structure was most clearly visible in mummified cells (1000 µM of 4HR), both those incubated for 24 h and those incubated for 16 months (Figure 2G,H). Since the two dark lines in TEM images of cells represent a visualization of the osmium-stained lipid bilayer, the disruption of this structure and the appearance of a third line reflects the effect of 4HR on the bilayer lipids. The thickness of the membrane increased by approximately one and a half to two times when the third dark line appeared. The mechanism of this phenomenon can be established using molecular modeling methods.

### 3.3. Modeling of Membranes with 4HR

Based on microbiological and microscopic data, we continued to investigate the membranes of Gram-negative bacteria *E. coli* by modeling the membranes with 4HR using classical molecular dynamics. It was used to explain the patterns identified in the experiments and answer the questions posed in this study. In particular, we were interested in the structural response of cell membrane compartments to the effects of 4HR, and the potential for damage and stretching of membranes.

**Figure 3:**
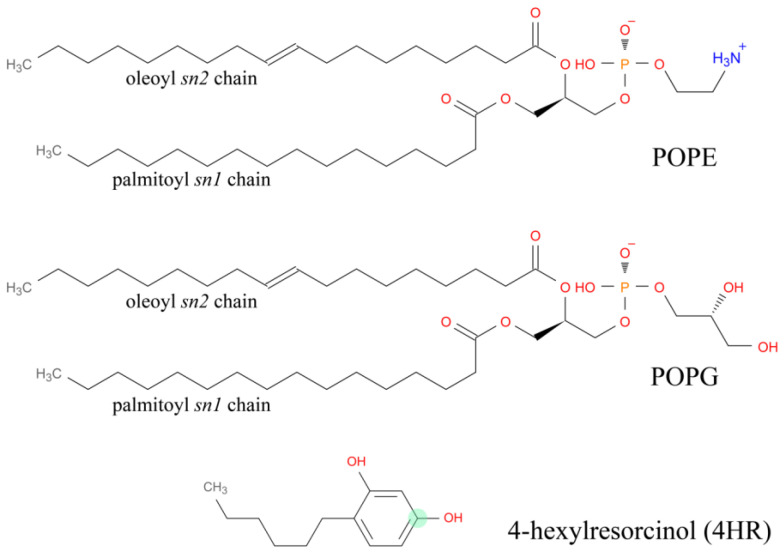
Structure of the membrane phospholipids POPE and POPG and the autoregulator 4HR. To construct Voronoi diagrams (see below), one of the lipid atoms must be selected; for phospholipids, the phosphorus atoms are selected; for 4HR, we selected the carbon atom in the resorcinol ring, which is highlighted in the 4HR image by the green circle.

In choosing the membrane, we relied on the capacity and simplicity of the Gram-negative membrane model that simulates *E. coli* membranes with acceptable accuracy. Therefore, for MD simulations, a universal membrane model POPE (1-palmitoyl-2-oleoyl-sn-glycero-3-phosphoethanolamine)/POPG (1-palmitoyl-2-oleoyl-sn-glycerol-3-phosphoglycerol) of Gram-negative bacteria *E. coli* was chosen. It contains two types of lipids, namely, POPE and POPG in a 3:1 ratio (Figure 3).

To ensure accurate comparison of theoretical and experimental data, the number of 4HR molecules near model membrane was set to correspond to the autoregulator concentrations used in the experiments. It was assumed that 4HR adsorbed on bacterial cells from the solution fairly quickly, and the number of 4HR molecules per 1 nm^2^ of cell membrane was calculated in each experimental variant. Then taking into account these data and the redistribution of 4HR to the outer and inner membranes, as well as the cell wall peptidoglycan, the correspondence of the experimental concentrations to the 4HR:lipid ratios in the simulation boxes were determined. Thus, 4HR concentrations in experiments of 10, 100, 200 and 1000 µM corresponded to 4HR:lipid ratios of 0.24:1, 1.2:1, 1.8:1 and 2.38:1 in the box. This correspondence demonstrated its full consistency with MD simulations previously conducted in other studies with 4HR [32].

### 3.4. The Mechanism of 4HR Interaction with Bacterial Cell Membranes Revealed by MD

**Figure 4:**
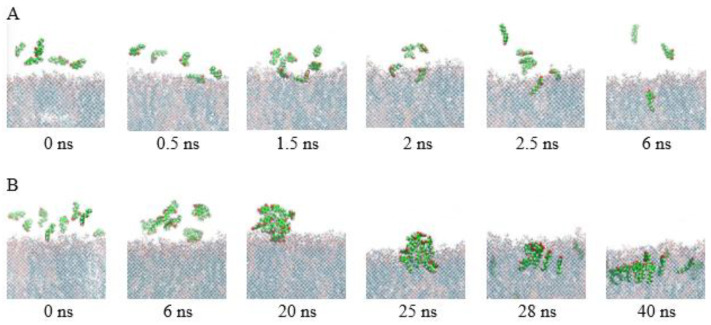
MD trajectory snapshots illustrating the process of penetration of single 4HR molecules into the membrane (**A**), formation of clusters and micelles of 4HR near membrane, penetration of micelles into the membrane and their dissolution in the membrane (**B**). POPE are blue, POPG are pink, 4HR are green with red oxygen atoms. Phospholipids are shown as transparent, while 4HR molecules are seen as bright using the VDW drawing method version 1.9.2 [43].

Experiments have shown that adding alcoholic solutions of 4HR to aqueous bacterial suspensions results in the formation of a micellar solution of the autoregulator. However, this does not lead to any complications in its effects on cells. We examined the interaction of single 4HR molecules and micelles of 4HR molecules that appear in experimental solutions with membranes. Simulations showed that 4HR penetrated the membrane and eventually distributed at the interface between the hydrophilic head groups and the hydrophobic core of the bilayer. This behavior was characteristic of both single molecules and micelles. (Figure 4). Initially (at extremely fast times of up to 6 ns), the following processes occur simultaneously and rapidly on the membrane surface: penetration of individual molecules into the membrane, adsorption of 4HR on the surface of membrane, and micelle formation of 4HR. The micelles formed by 4HR are single-layered, with resorcinol rings exposed on the outside and hydrophobic hydrocarbon tails (6 carbon atoms) on the inside (Figure 4B, 20 ns). As the simulation progresses, larger micelles also penetrate the membrane (25 ns) and begin to distribute within the membrane (40 ns). After penetrating into the membrane, 4HR micelles dissociate, and individual molecules are distributed across the membrane. 4HR molecules are also able to pass through the membrane. As was established, the Gibbs free energy barrier value obtained by umbrella sampling studies of 4HR migration through the POPE/POPG bilayer is (4.54 ± 0.60) kcal/mol [32].

**Figure 5:**
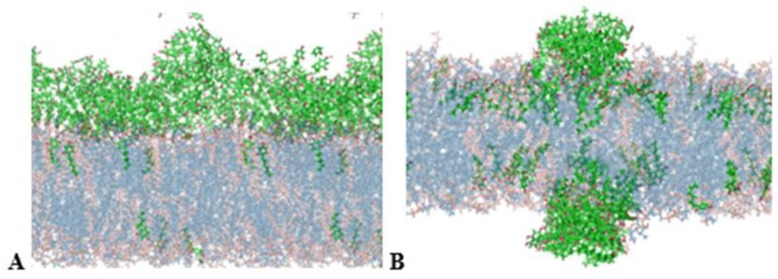
MD trajectory snapshots illustrating the process of adsorption of 4HR molecules at its high concentration in solution > 200 µM (**A**), violation of membrane smoothness when large 4HR micelles are embedded into it (**B**). POPE are blue, POPG are pink, 4HR are green with red oxygen atoms. Phospholipids are shown as transparent, while 4HR molecules are seen as bright using the VDW drawing method.

When the 4HR concentration in solution exceeds 200 µM (4HR:lipid ratio > 1.8:1), its amount in the cell becomes so great that micelles cover the cell membranes in a thick layer, partially penetrating into the lipid head regions (Figure 5A). Modeling showed that at such high concentrations, 4HR exists only as agglomerates, not individual molecules. The agglomerates disrupt the membrane’s smoothness, protruding beyond its boundaries above and below the lipid head groups (Figure 5B). To accommodate all of the 4HR at such a high concentration, the membrane must be extremely stretched. Such stresses are easily created in a computational experiment. However, for a living cell, such stresses may lead to cell death. Therefore, in experimental variants with high 4HR concentrations, the formation of agglomerates or even micellar arrays within cell membranes leads to cell death through mummification. In addition, in some cells, such stresses cause membrane ruptures, leading to lysis. Moreover, as microbiological experiments show, the cell is unable to get rid of excess 4HR. Simulation confirms these data. MD shows that when the membrane is stretched by increasing the surface tension to 40 mN/m, it is capable of accommodating a significant number of 4HR molecules. Subsequent relaxation of the membrane at zero surface tension reveals the effect of trapping most 4HR molecules within it, with the exception of a few. Apparently, procedures for reactivating such cells by removing 4 HR are no longer useful, since the cells have already died, although they were not lysed but remained intact.

### 3.5. Determination of the 4HR Concentration Leading to Membrane Saturation

**Figure 6:**
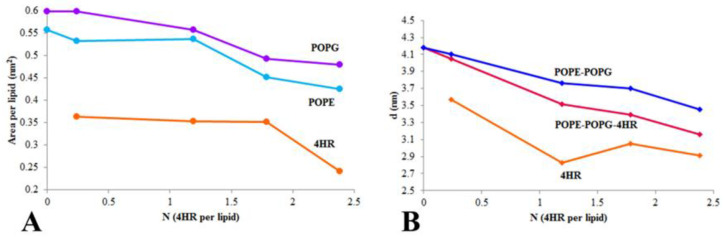
Effect of 4HR concentration on: (**A**)—Area per lipid (APL); (**B**)—Average leaflet thickness (LT). (**A**) Purple line—APL for POPG, ice blue—POPE, orange—4HR. (**B**) Red line—both phospholipids and 4HR are taken into account, blue—only phospholipids are taken into account, orange—only 4HR is taken into account. Calculations were performed using the Voronoi method.

The gradual incorporation of 4HR molecules into membranes with increasing concentration in solution should affect the average area occupied by one lipid molecule in the bilayer, the so-called “Area per lipid” (APL). Calculations of the change in area per lipid with increasing 4HR concentrations were performed using the Voronoi method [44,45]. The data were obtained by analyzing the last 200 ns of microsecond trajectories. It turned out that in the 4HR concentration range from 0 to 1.8:1 (from 0 to 200 µM in solution), APL decreased for both POPE (from 0.56 ± 0.01 nm^2^ to 0.45 ± 0.01 nm^2^) and POPG (from 0.60 ± 0.02 nm^2^ to 0.49 ± 0.03 nm^2^) (Figure 6A). At the same time, the area per 4HR molecule in the membrane remained unchanged and amounted to ~0.35 ± 0.05 nm^2^. A further increase in 4HR concentration no longer caused a decrease in the area per lipid for POPE and POPG, which indicated the saturation of the membrane with autoregulator molecules. However, the area per 4HR molecule began to decrease, illustrating the dense packing of the remaining 4HR molecules in the membrane without intercalation between phospholipids. Thus, this calculation showed that a 4HR concentration of 1.8:1 in the periodic box, corresponding to a concentration of 200 µM 4HR in solution, is the saturation concentration of membranes with autoregulator molecules.

The effect of 4HR concentration on membrane leaflet thickness was also calculated using the Voronoi method. Only those 4HR molecules that dissolved in the membrane were considered in the calculations. Leaflet thickness also decreased with increasing 4HR concentration, both when considering only POPE and POPG lipids and when including 4HR (Figure 6B). This was a consequence of lipid disordering in the membrane under the influence of 4HR.

### 3.6. Distribution of 4HR Micelles in Bacterial Cell Membranes Visualized by MD

**Figure 7:**
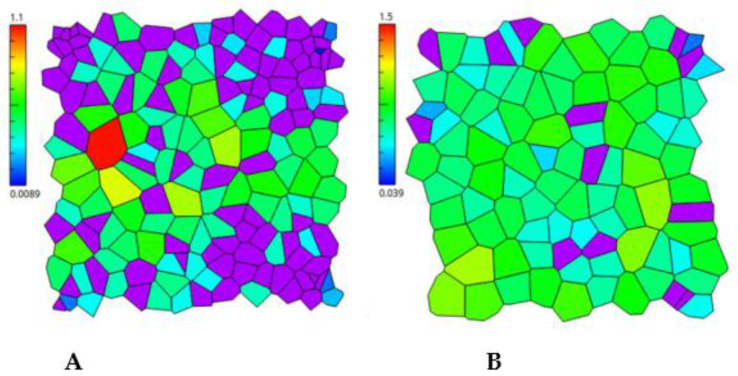
Voronoi diagrams for POPE/POPG membranes at 1.19:1 ratio of 4HR:lipid for 210 nanosecond (ns) time point. Areas per lipid (APL) for POPE and POPG molecules are shown in colors from red to blue according to the numerical value in nm (see corresponding scales). The areas of 4HR molecules are highlighted in purple. (**A**,**B**)—The upper and lower leaflets of the same membrane. The reference points relative to which the distributions were constructed were the phosphorus atoms of phospholipids and the carbon atom of 4HR, highlighted by the green circle in Figure 3.

The distribution of micelles within the membrane was studied in more detail at 4HR concentrations below 200 mM. These conditions are less critical than those described above. It was shown that 4HR micelles penetrate the membrane and are distributed unevenly within it. Moreover, the unevenness concerned not only the distribution within leaflet, but also between leaflets. During simulations lasting up to 1 μs, a jump of some molecules from one leaflet to another was observed. Figure 7 shows the Voronoi diagrams [44] for APL, obtained by AplVoro 3.3.3 [45], as 4HR micelles penetrate the membrane. They illustrate the distribution of 4HR molecules (purple) within leaflets (Figure 7) and between them (see the difference in the number of purple regions in Figure 7A,B). Taken together, these results indicate that 4HR molecules can readily penetrate the bilayer in aggregate form and loosen the lipid packing of the membrane. This can be reflected in changes in local membrane curvature and influence cell physiology.

**Figure 8:**
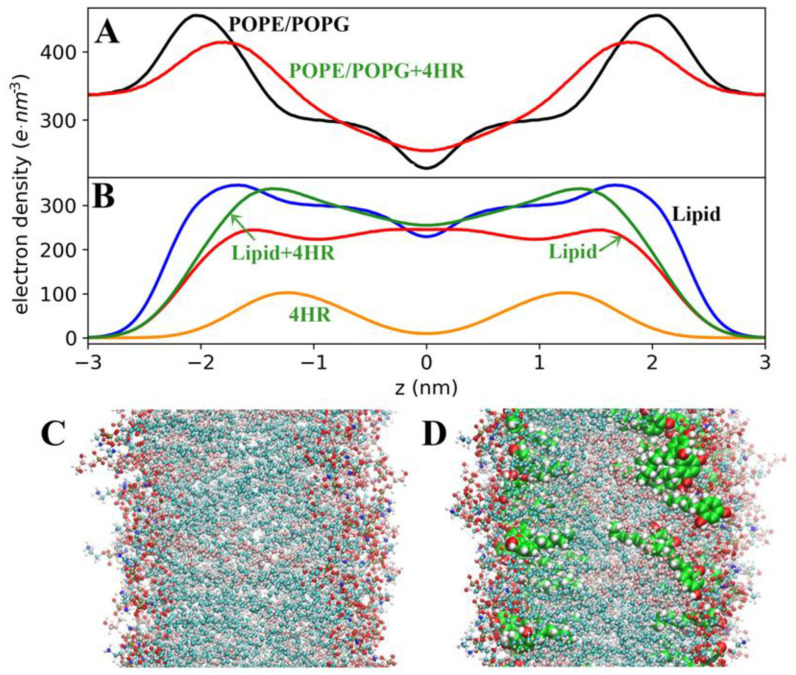
Electron densities profiles for membrane POPE/POPG (black inscriptions) and POPE/POPG/4HR (green inscription) with 1.8:1 ratio of 4HR to lipid obtained at 50–250 ns of MD trajectories. (**A**)—Total electron densities. (**B**)—Phospholipids and 4HR densities: phospholipids of POPE/POPG (blue), phospholipids of POPE/POPG/4HR (red), 4HR of POPE/POPG/4HR (orange), summed density of phospholipids with 4HR of POPE/POPG/4HR (green). (**C**,**D**)—side view of POPE/POPG and POPE/POPG/4HR bilayers. POPE carbons are ice blue, POPG carbons are pink, 4HR carbons are green, oxygens are red, hydrogens are white, nitrogens are blue, phosphorus atoms are gold. The VMD drawing method of phospholipids is CPK, while 4HR is VDW.

The location of 4HR molecules in the membrane thickness can be judged from the electron density profiles (Figure 8). For POPE/POPG bilayers without 4HR, the electron density maxima occur at a distance of 2 nm from the membrane center and are approximately 450 e·nm^−3^. The minimum electron density occurs at the membrane center and is approximately 230 e·nm^−3^, which corresponds to the literature data [46]. When 4HR molecules are incorporated into the bilayer, the curve changes due to the distribution of 4HR molecules between the lipid heads and the hydrophobic region of the membrane. The higher the concentration of 4HR, the more the total electron density profile of membranes changes (Figure 8A). At low 4HR concentrations, the effects are less noticeable, but identical, since the location of 4HR molecules in the membrane remains the same: their hexyl radicals are located deeper in the membrane than the resorcinol ring (Figure 8D). The 4HR electron density profiles form a smooth hump on each leaflet.

### 3.7. Redistribution of POPE and POPG Lipids Within the Membrane, Leading to the Appearance of a Third Peak on the Electron Density Profile of Phospholipids

MD simulations showed that the presence of 4HR inside the bilayer also leads to a displacement of lipids (Figure 8B,D). This is noticeable in the electron density profiles as a shift in the phospholipid density in Figure 8B (red curve) with the appearance of a third peak corresponding to the z = 0 coordinate. Also, in Figure 8D, compared to Figure 8C, it is clear that the “middle” band characteristic of bilayer patterns (the plane of lipid tail junction) disappears. The lipid tails of both leaflets partially intermix, which leads to the appearance of a third peak. This explains the appearance of a third, osmium-stained black band in TEM images of the outer membranes of *E. coli*, which bear the brunt of the impact of 4HR. As mentioned above, the effect of 4HR is manifested in all studied ratios, only with varying degrees of intensity. This is why we see this effect immediately in TEM images of mummified and resting cells, while at low concentrations it is not yet clearly visible after 24 h. However, in older cells, this third band effect is also visible at high concentrations, and it depends on the concentration.

### 3.8. Distribution of 4HR in Bacterial Cell Membrane Peptides Visualized by MD

An important part of the Gram-negative cell wall is the intermediate layer of peptidoglycan (PG), located between the outer and inner membranes. Atomic-scale simulations confirm that the cell wall models exhibit anisotropic elasticity, as observed experimentally. This anisotropy is due to the orthogonal orientation of glycan strands and peptide cross-links of PG.

**Figure 9:**
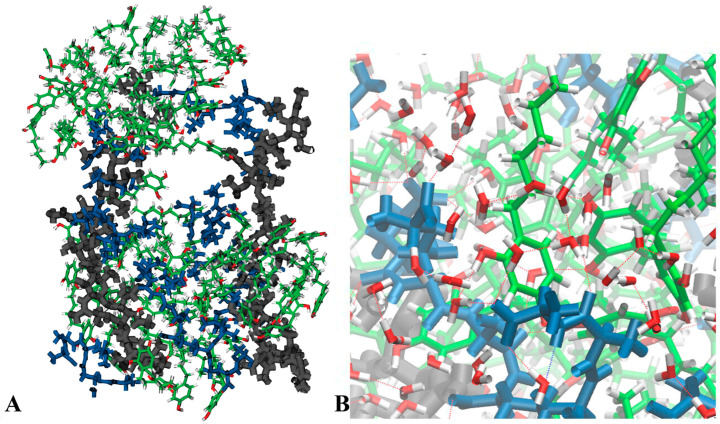
Absorption of 4HR molecules in peptidoglycan (**A**,**B**). General viewnot shown (**A**), hydrogen bonding of PG peptides with 4HR through water molecules. PG sugar-phosphate backbones are black, PG peptides are blue, 4HR are green with red oxygen atoms, water molecules are corners with red oxygen atoms and gray hydrogens, hydrogen bonds are red dots.

Based on the model studied by Gumbart et al. [47], we introduced 4HR molecules into the periodic box with PG. Simulations over 100 ns showed that 4HR molecules are absorbed into the PG (Figure 9). The figure shows a general view of the PG-4HR system. As in a water solution, 4HR assembles into micelles. However, unlike a water solution, the micelles assemble between the sugar strands and the peptide regions of PG. The mechanism of 4HR micelles binding to PG is shown in Figure 9B. One of the 4HR molecules in the micelle is shown in the foreground, linked to peptides by hydrogen bonds through water molecules. Thus, to link PG to 4HR micelles, chains of hydrogen bonds are formed: the peptides form hydrogen bonds with water, and the water molecules form hydrogen bonds with 4HR. It is clear from the simulations that the maximum concentration of 4HR that PG can accommodate is quite high. It is possible that the increase in the distance between the outer and inner membranes observed in long-stored DC obtained under the influence of 4HR is associated with the saturation of peptidoglycan with the autoinducer.

## 4. Discussion

The state of bacterial dormancy is a fundamental problem in microbiology, the study of which has been ongoing for over 100 years. As early as 1925, scientists posed the question of the differences between growth retardation and dormancy in bacteria and said that “Dormancy must be considered a factor in infection” [48]. Since then, the desire to understand all aspects of the transition of bacterial populations from active life to dormancy and back has not weakened. On the contrary, this problem comes to the fore, on the one hand, due to the danger of dormant bacteria to resist human immunity and drugs [49], and on the other hand, due to the ability to be useful in biotechnological industrial production [50] or collections of valuable microorganisms [51]. If scientists had a reliable tool or technology that would allow them to control the immersion of bacteria into dormancy and the emergence from it, this would revolutionize microbiological science. Perhaps this will someday occur as a result of the gradual accumulation of knowledge in this area.

Our group’s research has revealed certain patterns that occur in bacteria surviving unfavorable conditions in a dormant state [6]. The most successful of these studies were carried out at the intersection of sciences, utilizing the synergy of biophysical, biochemical, microbiological, and computational methods [9,31]. In this study, an attempt was made to involve the methods of classical MD in order to understand some issues of the mechanism of action of the chemical analog of autoinducer of anabiosis 4HR on cells of Gram-negative *E. coli* bacteria, that constantly arose during microbiological experiments.

1. 4HR penetrates the membrane of Gram-negative bacterial cells in the form of micelles 

The use of 4HR as an autoinducer of anabiosis in experiments with bacterial populations was hampered by its poor solubility in water. Alcohol solutions of the autoregulator were used to introduce 4HR into cell suspensions. However, when such solutions were transferred to an aqueous medium during work with bacterial suspensions, 4HR always formed micelles. However, a biological effect of the autoinducer on bacterial cells was still observed. The question arose as to how 4HR micelles act on bacteria and whether there is a difference in the biological effect of true or micellar 4HR solutions on cells. Simulations performed using classical MD methods in this study suggested that 4HR penetrates the Gram-negative cell membrane equally well, both as individual molecules and as micelles. Therefore, the use of true or micellar solutions in experimental work does not significantly affect cell regeneration. Upon entering the lipid bilayer, micelles immediately dissociate into individual molecules and distribute across the membrane. This distribution is extremely precise: the hydrocarbon tails (radicals) are aligned parallel to the lipid tails of POPE and POPG. However, as the 4HR concentration in the solution increases, the number of micelles can become so large that they cover the entire cell surface. Then, 4HR penetrates the cell en masse only in the form of micelles, which disrupt the cell’s smoothness and stretch it, leading to bacterial death either through membrane rupture (cell lysis) or through membrane overflow with agglomerates (cell mummification).

2. The distribution of 4HR in the membranes of Gram-negative bacterial cells and how this affects their structure

The distribution of micelles in the membrane is uneven, both within a leaflet and between them. This was clearly demonstrated in Voronoi diagrams for the area per lipid. Regions supersaturated with 4HR micelles can appear in the cell membrane. Thus, some cells die even when given small doses of 4HR simply because 4HR penetrates their membrane locally, at a high dose, in one place, causing membrane rupture.

The incorporation of 4HR into membranes redistributes the membrane’s electron density in a specific manner, independent of 4HR concentration. The concentration only influences the magnitude of the effect: the higher the concentration, the stronger the effect. The same occurs with the redistribution of the lipid heads and tails of POPE and POPG molecules within the membrane.

The lipid tails of both leaflets partially mix upon exposure to 4HR, which can result in a third peak in the electron density profiles and a third dark osmium-stained line in transmission electron microscopy images of the outer cell membrane. A third dark line also appears in the inner cell membrane, although it is more difficult to detect. The magnitude of the effect also depends on concentration; therefore, at high doses of 4HR, three dark lines in the outer or inner membrane become visible after a short incubation period. At lower doses, the effect develops more slowly. The appearance of an additional dark line increases the overall membrane size by 1.5–2 times, which is easily detected in TEM images.

It should be noted that this effect of altering membrane structure is characteristic of stressed cells, DCs, and mummified cells obtained under the influence of 4HR and has not yet been observed in similar forms obtained under natural conditions, such as during starvation.

3. The effect of 4HR on the cell wall peptide of Gram-negative bacterial cells

In addition to the outer and inner membranes of bacterial cells, 4HR also affects the middle layer of the cell wall—peptidoglycan. Peptidoglycan consists of polysaccharide chains cross-linked by peptide bridges. Its main function is to provide mechanical strength and protection against osmotic lysis. The peptide glycan layer in *E. coli* is usually determined to be from 2 to 8 nm thick, with the obtained values varying depending on the measurement method and sample preparation. Variations in thickness are explained by the fact that peptidoglycan is flexible and dynamic, can depend on the hydration state of the cell, and has a variable number of layers (unevenly distributed) over the entire cell surface [52]. Remodeling of peptidoglycan is one of the strategies for bacterial survival when the outer membrane is ruptured [53]. Simulations showed that 4HR freely penetrates the peptidoglycan layer, where it assembles into micelles between the sugar strands and peptide regions of the peptidoglycan. Moreover, hydrogen bond chains are formed to link the peptidoglycan structure to the 4HR micelles. DCs obtained under the influence of 4HR often exhibit an increase in the distance between the outer and inner membranes, which is possibly due to saturation of the peptidoglycan with autoinducer molecules.

4. Concentration effect of 4HR on the membranes of Gram-negative bacterial cells

Simulations of the change in the average area per lipid molecule in the bilayer membrane with increasing 4HR concentrations made it possible to determine the autoinducer concentration at which membrane saturation occurs. It was 1.8:1 in the calculation cell, which corresponds to a concentration of 200 µM (~40 μg/mL) 4HR in solution. These calculated data are well characterized by experimental data, which showed that this concentration is critical and causes a rapid transition of cells to a dormant state. The use of a smaller amount of 4HR causes the formation of cells that are resistant to stress, but do not resemble DCs in their general morphology, while higher doses of 4HR lead to consequences incompatible with life. Of course, the concentration determined in the calculations is an approximate guideline, not an exact value, since when modeling biological processes, it is impossible to take into account all the factors occurring in a real process. Laboratory experiments demonstrated that exposure to 4HR in the concentration range of (150–250) µM causes a rapid transition of various types of Gram-negative bacterial cells into a dormant state. Molecular modeling demonstrated that in this concentration range, cell membranes are completely saturated with 4HR molecules. Higher concentrations of 4HR may lead to membrane supersaturation and, consequently, the inability to perform their functions. Lower concentrations will not produce the desired effect. This finding should be considered as one link in the mechanism of cell transition to a dormant state, explaining the effect of 4HR only on cell membranes and not affecting the effect on other bacterial biopolymers. However, the good correlation between experimental and theoretical data confirms the correct approach to this issue. Explaining the formation of DCs in terms of membrane saturation with 4HR will allow for more effective and meaningful planning of future experiments.

## 5. Conclusions

This study answered all the important questions posed at the outset. A combination of experimental and computational methods allowed us to offer an explanation for how 4HR penetrates the membranes of Gram-negative bacterial cells, how it affects the lipid bilayer and peptide interlayer, and why a certain 4HR concentration is a turning point separating the formation of stressed, dormant, and mummified cells. However, this study is only the beginning of a long journey. Many questions remain about the mechanisms of bacterial dormancy induced by 4HR or prolonged starvation. It is also important to understand how cells awaken after prolonged dormancy or how dormant cells induced by 4HR can be reanimated. The use of nonequilibrium in silico studies and more complex membrane models in the future will allow for more comprehensive results. The ability to study such processes in silico opens up great prospects for microbiology.

## Figures and Tables

**Table 1:** Model systems with POPE/POPG membranes. The POPE:POPG ratio is 3:1.

Number, Membrane/Box	4HR:Lipid Ratio	Water:Lipid Ratio	Total Atoms
1 membrane POPE/POPG	0:1	48.6	45,676
2 membrane POPE/POPG + 4HR	0.24:1	47.3	45,668
3 membrane POPE/POPG + 4HR	1.2:1	40.4	44,727
4 membrane POPE/POPG + 4HR	1.8:1	34.5	43,330
5 membrane POPE/POPG + 4HR	2.38:1	27.7	41,531

**Table 2:** Changes in the viability titer of *E. coli* Top10 cells in populations with different concentrations of 4HR during long-term storage.

4HR Concentration, µM	Viable Cell Titer (Cells/mL) After Incubation with 4HR for a Specified Time				
	40 min	1 Day	7 Days	1 Month	16 Months
Control (0)	(2.2 ± 0.3) ×10^9^	(2.0 ± 0.3) ×10^9^	(9.3 ± 0.4) ×10^8^	(6.1 ± 0.4) ×10^7^	(4.7 ± 0.4) ×10^6^
10	(2.3 ± 0.2) ×10^9^	(1.7 ± 0.2) ×10^9^	(1.2 ± 0.1) ×10^9^	(3.5 ± 0.4) ×10^8^	(4.9 ± 0.2) ×10^7^
100	(2.0 ± 0.3) ×10^9^	(1.1 ± 0.3) ×10^9^	(6.6 ± 0.3) ×10^8^	(3.8 ± 0.3) ×10^7^	(2.1± 0.1) ×10^7^
200	(6.5 ± 0.4) ×10^8^	(3.1 ± 0.4) ×10^7^	(5.9 ± 0.4) ×10^5^	0 After reactivation (1.5 ± 0.1) ×10^4^	0 After reactivation 0
1000	0	0	0	0	0

**Table 3:** Stress resistance of long-term stored DCs of *E. coli* Top10 in populations with different concentrations of 4HR.

4HR Concentration, µM	Viable Cell Titer, in % of the Initial		
	After 16 Months of Incubation *	After Heat Shock 50 °C 20 min **	After Heat Shock 60 °C 20 min **
Control (0)	0.21	17.1	0.02
10	2.1	41.9	0.23
100	1.1	36.6	0.14

* The initial cell titer was taken as their number in the population after 40 min of exposure to 4HR. ** DCs stored for 16 months were tested for stress resistance.

## Data Availability

The original contributions presented in this study are included in the article. Further inquiries can be directed to the corresponding authors.
